# Mechanisms of *CFTR* Functional Variants That Impair Regulated Bicarbonate Permeation and Increase Risk for Pancreatitis but Not for Cystic Fibrosis

**DOI:** 10.1371/journal.pgen.1004376

**Published:** 2014-07-17

**Authors:** Jessica LaRusch, Jinsei Jung, Ignacio J. General, Michele D. Lewis, Hyun Woo Park, Randall E. Brand, Andres Gelrud, Michelle A. Anderson, Peter A. Banks, Darwin Conwell, Christopher Lawrence, Joseph Romagnuolo, John Baillie, Samer Alkaade, Gregory Cote, Timothy B. Gardner, Stephen T. Amann, Adam Slivka, Bimaljit Sandhu, Amy Aloe, Michelle L. Kienholz, Dhiraj Yadav, M. Michael Barmada, Ivet Bahar, Min Goo Lee, David C. Whitcomb, the North American Pancreatitis Study Group

**Affiliations:** 1 Department of Medicine, Division of Gastroenterology, Hepatology and Nutrition, University of Pittsburgh, Pittsburgh, Pennsylvania, United States of America; 2 Department of Pharmacology and Brain Korea 21 Plus Project for Medical Science, Yonsei University College of Medicine, Seoul, Korea; 3 Department of Computational & Systems Biology, University of Pittsburgh, Pittsburgh, Pennsylvania, United States of America; 4 Division of Gastroenterology and Hepatology, Mayo Clinic, Jacksonville, Florida, United States of America; 5 Department of Medicine, University of Michigan, Ann Arbor, Michigan, United States of America; 6 Division of Gastroenterology, Brigham and Women's Hospital, Boston, Massachusetts, United States of America; 7 Digestive Disease Center, Medical University of South Carolina, Charleston, South Carolina, United States of America; 8 Department of Medicine, Duke University Medical Center, Durham, North Carolina, United States of America; 9 Department of Internal Medicine, St. Louis University School of Medicine, St Louis, Missouri, United States of America; 10 Department of Medicine, Indiana University School of Medicine, Indianapolis, Indiana, United States of America; 11 Dartmouth-Hitchcock Medical Center, Hanover, New Hampshire, United States of America; 12 North Mississippi Medical Center, Tupelo, Mississippi, United States of America; 13 Division of Gastroenterology, Hepatology and Nutrition, Virginia Commonwealth University Medical Center, Richmond, Virginia, United States of America; 14 Department of Human Genetics, University of Pittsburgh, Pittsburgh, Pennsylvania, United States of America; 15 Department of Cell Biology and Molecular Physiology, University of Pittsburgh, Pittsburgh, Pennsylvania, United States of America; Emory University School of Medicine, United States of Americs

## Abstract

CFTR is a dynamically regulated anion channel. Intracellular WNK1-SPAK activation causes CFTR to change permeability and conductance characteristics from a chloride-preferring to bicarbonate-preferring channel through unknown mechanisms. Two severe CFTR mutations (*CFTR^sev^*) cause complete loss of CFTR function and result in cystic fibrosis (CF), a severe genetic disorder affecting sweat glands, nasal sinuses, lungs, pancreas, liver, intestines, and male reproductive system. We hypothesize that those *CFTR* mutations that disrupt the WNK1-SPAK activation mechanisms cause a selective, bicarbonate defect in channel function (*CFTR^BD^*) affecting organs that utilize CFTR for bicarbonate secretion (e.g. the pancreas, nasal sinus, vas deferens) but do not cause typical CF. To understand the structural and functional requirements of the CFTR bicarbonate-preferring channel, we (a) screened 984 well-phenotyped pancreatitis cases for candidate *CFTR^BD^* mutations from among 81 previously described *CFTR* variants; (b) conducted electrophysiology studies on clones of variants found in pancreatitis but not CF; (c) computationally constructed a new, complete structural model of CFTR for molecular dynamics simulation of wild-type and mutant variants; and (d) tested the newly defined *CFTR^BD^* variants for disease in non-pancreas organs utilizing CFTR for bicarbonate secretion. Nine variants (*CFTR* R74Q, R75Q, R117H, R170H, L967S, L997F, D1152H, S1235R, and D1270N) not associated with typical CF were associated with pancreatitis (OR 1.5, p = 0.002). Clones expressed in HEK 293T cells had normal chloride but not bicarbonate permeability and conductance with WNK1-SPAK activation. Molecular dynamics simulations suggest physical restriction of the CFTR channel and altered dynamic channel regulation. Comparing pancreatitis patients and controls, *CFTR^BD^* increased risk for rhinosinusitis (OR 2.3, p<0.005) and male infertility (OR 395, p<<0.0001). WNK1-SPAK pathway-activated increases in CFTR bicarbonate permeability are altered by *CFTR^BD^* variants through multiple mechanisms. *CFTR^BD^* variants are associated with clinically significant disorders of the pancreas, sinuses, and male reproductive system.

## Author Summary

Genetic disorders of ion channels can affect the body's ability to function properly in many ways. CFTR, an ion channel regulating movement of chloride and bicarbonate across cell membranes, is important for absorbing and secreting fluids. If the gene responsible for the CFTR channel is mutated severely, the result is cystic fibrosis, a hereditary disorder in which the patient develops thick mucus, especially in the lungs, as well as scarring (fibrosis) in the pancreas. Cystic fibrosis also affects the sweat glands, nasal sinuses, intestines, liver, and male reproductive system. Mutations to the CFTR gene that do not cause cystic fibrosis have been considered benign. However, we discovered 9 CFTR mutations that do not cause cystic fibrosis but do cause inflammation and scarring of the pancreas (chronic pancreatitis). These mutant CFTR channels secrete chloride, which is important in the sweat glands, lungs, and intestines, but not bicarbonate, which is important in the pancreas, sinuses, and male reproductive tract. We found patients with any of these 9 mutations had chronic pancreatitis, and often sinus infections, and male infertility, but not other symptoms of cystic fibrosis. Our computer models and data will help researchers develop better drugs and help physicians treating patients with chronic pancreatitis.

## Introduction

The cystic fibrosis transmembrane conductance regulator (CFTR, GenBank Accession: AH006034.1) is an ATP-binding cassette (ABC) transporter-type protein localized to the apical plasma membrane of epithelial cells. It differs from other ABC transporters in that it acts as a regulated anion channel rather than a transporter [1]. When the channel is open, anions move across the membrane down their electrochemical potential gradient, resulting in fluid and electrolyte secretion or absorption.

The CFTR molecule has been intensely studied because mutations in the CFTR gene are associated with cystic fibrosis (CF, OMIM #219700), the most common life-threatening genetic disorder among populations of Northern European ancestry[2], [3]. However, the clinical features of CF and CFTR-related disorders are variable, and laboratory studies of CFTR regulation, its biophysical properties and molecular mechanisms of (dys)function have been challenging due to the complexity of the regulatory mechanisms and the dynamic flexibility of various structural domains (see recent reviews [4], [5]).

Cystic fibrosis is an autosomal recessive syndrome usually caused by inheriting two *CFTR* mutations that eliminate effective chloride conductance (*CFTR^CF^*)[2], [3]. Although nearly 2000 CFTR variants have been described (http://www.genet.sickkids.on.ca), the majority of CF cases are associated the *CFTR* 508F-del mutation as a homozygous genotype or in combination with another severe CF-associated mutation (*CFTR^CF^*/*CFTR^CF^*) that together result in minimal CFTR function. Thus, most research has focused on the regulation of chloride conductance, and dynamic modeling of the first of two nucleotide-binding domains (NBD1), which normally contains F508 [4], [5]. Based on numerous studies, three conformations have been described for the molecule as an anion channel: a closed state, an open state, and an open-ready state [4]. However, the relative permeability/conductance ratios of chloride and bicarbonate are variable [6] and may be dynamically regulated [7], suggesting that conformational changes induced by point mutations in the channel or in the permeability pore may alter ion permeation properties of CFTR.

Diagnosis of CF is based on a combination of phenotypic features, family history, functional tests and/or the identification *CFTR^CF^* variants on both alleles[8], [9]. Organ dysfunctions begin *in utero* and include chronic pancreatitis, meconium ileus, and congenital bilateral agenesis of the vas deferens. Progressive sinorespiratory dysfunction develops in childhood due to bacterial infections, inflammation, and scarring, and male infertility is recognized in adulthood. Disease severity and complexity is modified by other genes[10]–[12], environmental factors[13], and mild-variable *CFTR* variants [3], [14]. Mild CF phenotypes, CFTR-related disorders limited to a single organ, are associated with non-*CFTR^CF^* variants with residual channel function, classified as mild-variable variants (*CFTR* ^m-v^) [2], [9], [15].

*CFTR^sev^* and *CFTR^m-v^* variants are associated with recurrent acute pancreatitis and chronic pancreatitis [16]–[19]. Recently, we reported that the variant *CFTR* R75Q, which was previously classified as benign, is associated with familial and sporadic chronic pancreatitis, either with another *CFTR* variant (recessive) or with the serine protease inhibitor, Kazal Type 1 (*SPINK1*) N34S high-risk haplotype (complex genotype)[18]. Patch-clamp studies of CFTR R75Q clones under standard conditions demonstrated normal chloride conductance but a selective disruption in bicarbonate conductance[18]. Thus, *CFTR* R75Q causes selective bicarbonate defective (*CFTR^BD^*) conductance and is associated with chronic pancreatitis but not CF[18]. It is not known if other *CFTR* variants share this phenotypic feature, whether the defect is associated with the channel function under all or special conditions, or if other mechanism(s) underlying these observation.

Independently, we demonstrated that CFTR bicarbonate (HCO_3_^−^) permeability increases through WNK1-SPAK signaling pathway activation [20]. WNK1 is member of the “with-no-K” (Lys) kinases that serves as a sensor of osmolality, chloride concentration, and other factors within cells and respond by activating additional kinases linked to a variety of ion channels and exchanges, including CFTR [20]–[22]. In cell-based models, low intracellular chloride concentrations ([Cl^−^]_i_) result in WNK1-mediated SPAK activation that strongly increases CFTR HCO_3_^−^ permeability in CFTR-transfected HEK 293T, PANC1, and guinea pig pancreatic duct cells, making CFTR primarily an HCO_3_^−^ channel [20]. The structural and dynamic mechanisms of this phenomenon are unknown.

We hypothesized that CFTR variants that disrupt the WNK1-SPAK-associated increase in bicarbonate permeability will increase the risk of pancreatitis and affect other organs in which CFTR is used for bicarbonate secretion. To test this hypothesis and to gain insight into potential mechanisms, we adopted a multidisciplinary approach. First, to identify candidate *CFTR^BD^* variants, we conducted a systematic review of the literature to compile *CFTR* variants that have been reported at least twice in previous chronic pancreatitis case-control genetic studies, plus common *CFTR^CF^* variants. Second, using this panel of 81 *CFTR* variants (**Table S1** in the Supplementary Material), we genotyped the deeply phenotyped North American Pancreatitis Study 2 (NAPS2) subjects[23] to identify candidate *CFTR^BD^* variants that were also present in our cases and controls (43 of them, listed in **Table 1**). Third, to determine if *CFTR^BD^* variants are associated with altered WNK1-SPAK pathway-stimulated CFTR bicarbonate permeability, we generated plasmids containing the candidate *CFTR^BD^* variants selected from the NAPS2 study and expressed them in HEK-293T cells for electrophysiological analysis. Fourth, to gain insight into the molecular mechanisms of *CFTR^BD^* dysfunction, we performed molecular dynamics (MD) simulations based on homology-modeled structures of ABC transporters and examined the effect of *CFTR^BD^* variants on the structure and dynamics of the channel. Finally, to determine if *CFTR* ^BD^ variants are associated with disease in non-pancreatic tissues, we used the phenotyping criteria for sinusitis and male infertility for the NAPS2 cases and controls.

**Table 1:** Analysis of *CFTR* and *SPINK1* variants in cases and controls.

CFTR variant	%Cases	%Uctrls	OR	p-value	%Cases w/N34S	OR w/N34S	p-value w/N34S
**CF/BD or BD/BD**	2.5	0.1	**31.9**	**<0.0001**	5.5	7.46	0.12
**All CF**	8.7	3.3	**2.76**	**<0.0001**	16.4	**5.65**	**<0.0001**
**F508del^CF^**	6.9	3.1	**2.32**	**<0.0001**	14.5	**5.13**	**<0.0001**
IVS8T5**^CF^	9.9	8.2	**1.24**	0.079	10.9	1.37	0.47
**2789+5G>A^CF^**	0.3	0.0		**0.028**	0.0		
**3849+10kbC>T^CF^**	0.3	0.0		**0.028**	0.0		
**N1303K^CF^**	0.3	0.0		**0.027**	0.0		
**621+1G>T^CF^**	0.1	0.0		0.13	1.8		**<0.0001**
**2184delA^CF^**	0.1	0.0		0.13	0.0		
**3120+1G>A^CF^**	0.1	0.0		0.13	0.0		
**G551D^CF^**	0.2	0.1	2.50	0.20	0.0	0.00	0.83
**W1282X^CF^**	0.2	0.1	2.50	0.20	0.0	0.00	0.83
**G542X^CF^**	0.2	0.0		0.059	0.0		
**R1162X^CF^**	0.1	0.0		0.13	0.0		
**2183AA>G^CF^**	0.0	0.1		0.17	0.0	0.00	0.83
**All BD**	14.2	9.8	**1.50**	**0.002**	25.5	**4.63**	**<0.0001**
**R75Q^BD^**	6.3	6.2	1.02	0.30	16.4	**2.97**	**0.003**
**S1235R^BD^**	2.4	1.4	1.69	0.052	1.8	1.30	0.80
**R117H^CF/BD^**	2.3	0.7	**3.49**	**0.0007**	5.5	**8.74**	**0.0002**
**L967S^BD^**	1.1	0.2	**6.87**	**0.002**	1.8	**11.17**	**0.014**
**L997F^BD^**	0.8	1.0	0.82	0.26	1.8	1.84	0.55
**D1152H^BD^**	0.4	0.0		**0.014**	0.0		
**D1270N^BD^**	0.3	0.2	1.25	0.29	0.0	0.00	0.71
**R170H^BD^**	0.3	0.0		**0.028**	0.0		
**R74Q^BD^**	0.3	0.1	3.02	0.17	1.8	**21.15**	**0.002**
**Other**							
**M470V**	76.1	74.2	1.11	0.14	70.9	0.85	0.59
**T854T**	57.3	57.8	0.98	0.29	45.5	0.61	0.071
**Q1463Q**	39.6	39.5	1.01	0.30	40.0	1.02	0.94
1001+11C>T*	13.4	10.9	1.27	0.016	14.5	1.40	0.42
**125G>C**	10.3	9.7	1.07	0.26	12.7	1.36	0.45
**P1290P**	7.6	7.9	0.95	0.28	7.3	0.91	0.86
**1716G>A**	4.5	4.1	1.10	0.26	1.8	0.43	0.39
**R668C**	1.0	1.4	0.72	0.19	0.0	0.00	0.38
**G576A**	0.7	1.2	0.58	0.11	0.0	0.00	0.41
**F508C**	0.5	0.3	1.58	0.21	0.0	0.00	0.67
**R1162L**	0.5	0.5	1.13	0.29	1.8	4.03	0.17
**I1027T**	0.5	0.3	1.99	0.17	0.0	0.00	0.70
**R31C**	0.3	0.7	0.42	0.088	0.0	0.00	0.52
**I148T**	0.3	0.4	0.75	0.27	0.0	0.00	0.63
**R297Q**	0.3	0.2	1.89	0.21	0.0	0.00	0.76
**R74W**	0.2	0.2	0.85	0.29	0.0	0.00	0.71
**F1052V**	0.1	0.2	0.63	0.27	0.0	0.00	0.76
**I807M**	0.1	0.1	1.26	0.30	0.0	0.00	0.83
**R258G**	0.1	0.1	1.26	0.30	0.0	0.00	0.83
**G1069R**	0.1	0.0		0.13	0.0		
**V201M**	0.0	0.1		0.17	0.0	0.00	0.83

Of the 81 *CFTR* mutations tested in the cohort, 43 were observed at least once in cases or controls. Data shown for *CFTR* variant alone and, in cases, with a concurrent heterozygous variant in *SPINK1* N34S. *1001+11C>T is in linkage disequilibrium with F508del, risk calculation includes only 1001+11C>T not *in cis* with F508del. Blank cells indicate undefined (e.g. x÷0) **IVS8 T5 is reported but causes CF only when *in cis* with either R117H or IVS8 TG12or13. Intronic mutations are reported in standard nomenclature “####+/−##N>N” except IVS8-T5 (1210-12T[5]).

These studies revealed at least 9 *CFTR^BD^* variants. We found that the WNK1-SPAK pathway that enhances CFTR bicarbonate permeability/conductance compared with chloride conductance in HEK-293T cells is altered by *CFTR^BD^* variants. The examination of MD trajectories suggests at least two potential mechanisms of channel dysfunction. Phenotype-genotype studies in humans demonstrated that *CFTR^BD^* variants are also associated with disorders of the pancreas, sinuses, and male reproductive systems.

## Results

### CFTR genotyping in pancreatitis patients and controls

We genotyped 984 well-phenotyped cases of pancreatitis from NAPS2 for 81 *CFTR* variants, including common CF mutations and variants previously reported in at least two subjects with pancreatitis but not CF. Common tag-SNPs at the *CFTR* locus were previously excluded in a pancreatitis genome-wide association study (all p values ≥0.01) [24], suggesting that the missing heritability and predicted dysfunction was primarily associated with multiple rare variants. *SPINK1* N34S was also genotyped to determine complex risk [18]. Only *SPINK1* N34S heterozygotes were used for trans-heterozygote analysis with *CFTR*, since homozygous *SPINK1* N34S is sufficient to cause pancreatitis.

Of 43 *CFTR* variants identified in the NAPS2 cohort (**Table 1**), nine not associated with typical CF but reported in patients with pancreatitis[25]–[29] were of particular interest: R74Q, R75Q, R117H (*CFTR^m^* ^-v^ only when *in cis* with IVS8-T5[30]; R117H*T5), R170H, L967S, L997F, D1152H, S1235R, and D1270N. These were either independently associated with disease, were found in subjects with *SPINK1* N34S as a complex high-risk trans-heterozygous genotype or had predicted clinical relevance based on prior reports or their location on the CFTR molecule. Taken together, these nine *CFTR^BD^* variants were found more commonly in cases (14.2%) than controls (9.8%) (OR 1.5, p = 0.002) (**Table 1**).

As expected, *CFTR* variants associated with typical CF were also identified in more cases than controls (8.7% cases, 3.3% controls; OR 2.8, p<0.0001). Other candidate *CFTR* variants, including I148T, M470V, T854T, Q1463Q and the “5T” allele, were either rare or were not associated with pancreatitis in our cohort (**Table 1**). A total of 189 cases (19.8%) carried one or more *CFTR* variants of any kind (controls 13.0%, p<0.0001, OR 1.6, 95% C.I 1.3–2.0): 38% of these mutations were *CFTR^CF^* variants, while the remaining were *CFTR^BD^* variants (62%). Twenty-five cases and no controls carried multiple mutations in *CFTR*. Twenty-five cases carried trans-heterozygous mutations in both *CFTR* and *SPINK1 (N34S)*, including five patients with three or more mutations (**Table 2**).

**Table 2:** CFTR variants in subjects with chronic rhinosinusitis or male infertility (age >30 years).

Rhinosinusitis		Yes	No	p-value	OR	CI
Controls		53 (10.2%)	468	-	-	-
Cases (all)		151 (15.9%)	798	0.002	1.67	1.19–2.38
0 *CFTR^BD^*		111	649	0.021	1.51	1.05–2.18
0 *CFTR^CF^*		111	649	0.021	1.51	1.05–2.18
1 *CFTR^CF^*		14	50	**0.011**	**2.47**	**1.18–4.91**
1 *CFTR^BD^*		23	78	**0.001**	**2.60**	**1.43–4.60**
	1 *CFTR^CF^* or 1 *CFTR^BD^*	37	128	**0.0001**	**2.55**	**1.55–4.15**
*CFTR^BD^/CFTR^BD^* or *CFTR^BD^/CFTR^CF^*		3	21	0.73	1.26	NS

Infertility		Yes	No	p-value	OR	CI
Controls		1 (0.4%)	160	-	-	-
Cases (all)		17 (4.2%)	390	0.03	7.14	1.10–300
0 *CFTR^BD^*		8	329	0.28	3.88	0.5–174
0 *CFTR^CF^*		8	329	0.28	3.88	0.5–174
1 *CFTR^CF^*		2	24	0.051	13.0	0.65–786
1 *CFTR^BD^*		2	35	0.090	8.99	0.46–541
	1 *CFTR^CF^* or 1 *CFTR^BD^*	4	59	**0.023**	**10.7**	**1.03–536**
*CFTR^BD^/CFTR^BD^* or *CFTR^BD^/CFTR^CF^*		5	2	**1.20E-07**	**303**	**23–15783**

**Top:** Chronic rhinosinusitis in NAPS2 controls and cases with 0, 1, or 2 CFTR mutations. **Bottom**. Self-reported prevalence of male infertility among males over 30 years of age. Odds ratios were calculated comparing *CFTR* carrier cases in each subcategory against all controls. Because *CFTR^CF^* and *CFTR^BD^* both affect bicarbonate conductance, we calculated the association and risk associated with the presence of either variant type (shaded).

Several candidates that were previously reported to be associated with pancreatitis or atypical CF were not replicated in the NAPS2 cohort. I148T was seen in three cases and one control, so an effect could not be detected or excluded; the *in cis* deletion mutation 3199del6 was not detected in any I148T carriers. The IVS8T5 variant was identified in 9.9% of cases and 8.2% of controls, which is not individually significant. There were six N34S/T5 trans-heterozygote controls and no cases, but the combined effect of the *SPINK1* N34S variant with IVS8T5 was not significantly higher than N34S alone. Four variants were identified in only one patient and no controls: CF mutations 2184delA, 3120+1G>A, R1162X, and mutation of varying clinical consequence, G1069R.

### Functional assays on CFTR variants

**Figure 1:**
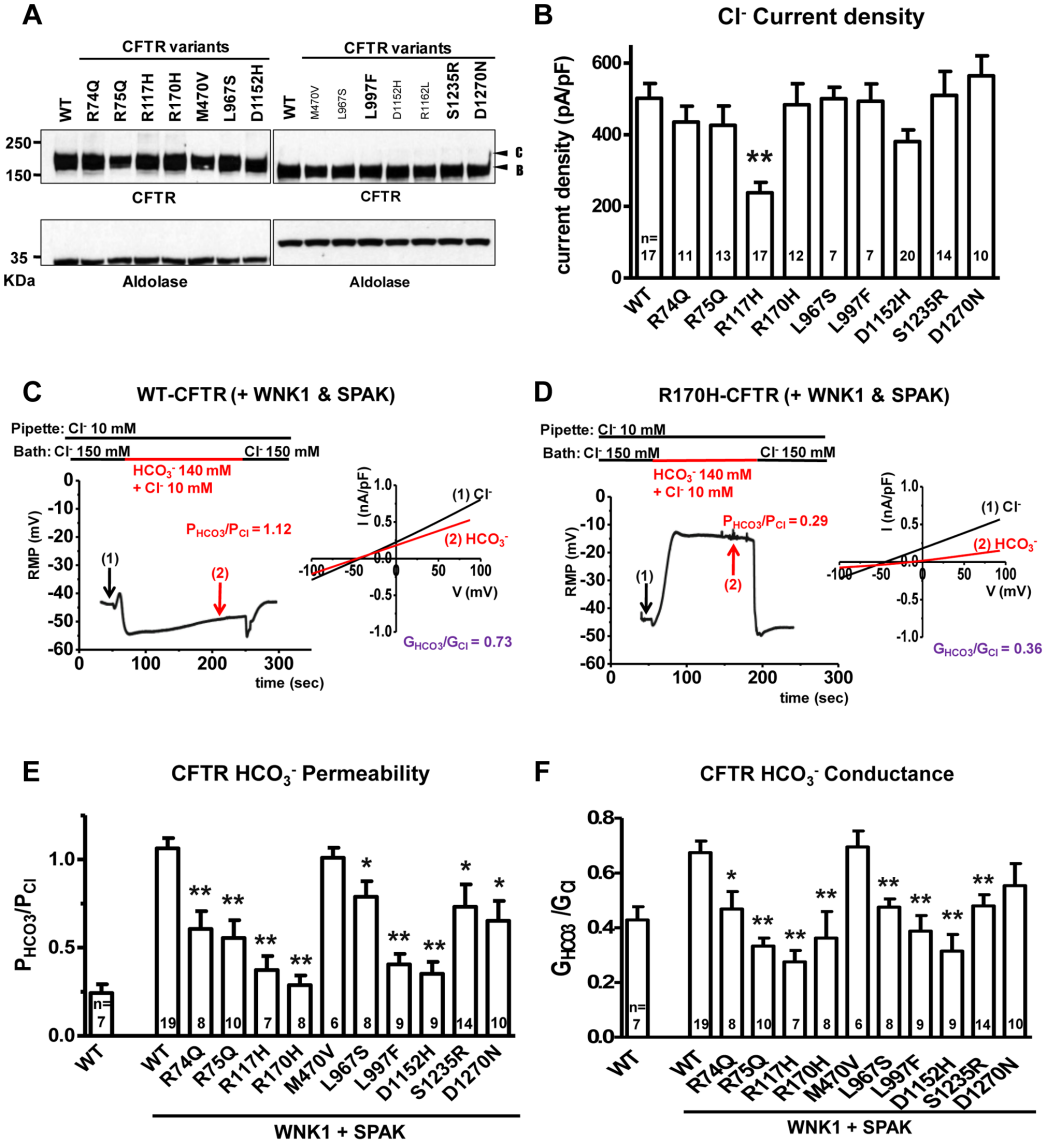
Functional characteristics of the nine *CFTR^BD^* variants. Panel **a**. Wild-type (WT) and variant CFTR proteins were expressed in HEK 293T cells and immunoblotted with anti-CFTR and anti-Aldolase antibodies. Replicate lanes are in small font. Band B, expected size of immature ER core-glycosylated CFTR; band C, mature complex-glycosylated CFTR. Panel **b**. Whole-cell Cl^−^ currents were measured in WT and variant CFTR-expressing HEK 293T cells, as described in Methods. Panel **c**. Whole-cell currents of WT-CFTR were measured in HEK 293T cells co-expressed with WNK1 and SPAK using patch pipette contained a low concentration of Cl^−^ (10 mM). A representative trace of reversal potential measurement is shown in the left panel. The permeability ratio P_HCO3_/P_Cl_ was calculated according to the Goldman-Hodgkin-Katz equation. I–V relationships at the indicated points are presented in the accompanying graph. The conductance ratio G_HCO3_/G_Cl_ was calculated by measuring each outward current (i.e., slope between E_rev_ and E_rev+25_ mV). RMP, resting membrane potential. Panel **d**. Whole-cell currents of R170H-CFTR were measured in HEK 293T cells using the same protocol shown in panel **c**. Panel **e**. A summary of the P_HCO3_/P_Cl_ values obtained from WT-CFTR in the standard state (left) compared to WT-CFTR and the nine *CFTR^BD^* variants with WNK1 + SPAK activation (right, underlined). Panel **f**. A summary of the G_HCO3_/G_Cl_ values in the standard state (left) with WNK1 + SPAK activation (right). Values throughout are means ± SEM. * p<0.05, **p<0.01: difference from WT in cells co-expressed with WNK1 and SPAK.

For our functional studies, we cloned the nine *CFTR* variants and confirmed that they had normal folding, glycosylation (**Figure 1a**) and chloride channel activities, except for R117H (**Figure 1b**). Because CFTR bicarbonate permeability is dynamically increased through [Cl^−^]_i_-sensitive WNK1-SPAK signaling pathway activation[20], we tested this in HEK 293T cells[20] using whole-cell current measurements by replacing 150 mM extracellular Cl^−^ with 140 mM HCO_3_^−^ and 10 mM Cl^−^. Representative traces for voltage and current measurements are presented in **Figures 1c, 1d**, and **S2**, and a summary of the indicated numbers of experiments is depicted in **Figure 1e and f**.

The bicarbonate permeability of CFTR in cells that do not overexpress WNK1 and SPAK was much smaller than that of chloride, with P_HCO3_/P_Cl_ = 0.24±0.05 (**Figure S1**). As reported previously [20], with WNK1 and SPAK co-expression and low [Cl^−^]_i_, the permeability of CFTR to bicarbonate increased and reached that of chloride, with P_HCO3/_P_Cl_ = 1.06±0.06 (**Figure 1c and 1e**). In contrast, CFTR P_HCO3/_P_Cl_ failed to increase in *CFTR* R170H (**Figure 1d**) and all of the candidate *CFTR^BD^* variants (**Figures 1e** **and S2**). Furthermore, all *CFTR^BD^* candidate variants lowered bicarbonate conductance (G_HCO3_/G_Cl_), which is an important metric determining apical bicarbonate efflux in CFTR-expressing epithelia (**Figure 1f**); the decrease was statistically significant for all variants except D1270N. Treatment with the CFTR inhibitor CFTR_inh_-172 (20 µM) inhibited >90% of the HCO_3_^−^ currents (**Figure S2**), indicating that CFTR mediates most of the HCO_3_^−^ currents observed in the present experiments.

To further evaluate the mechanism of bicarbonate conductance, we tested the hypothesis that the well-established CFTR channel blocker, CFTR_inh—_172[31]–[33] blocks HCO_3_^−^ current. We found that CFTR_inh_-172 (20 µM) inhibited >90% of the HCO_3_^−^ currents (Figure S2), indicating that with WNK1-SPAK activation, CFTR mediates most of the HCO_3_^−^ currents.

### Structural and dynamic modeling of CFTR wild-type and variants

**Figure 2:**
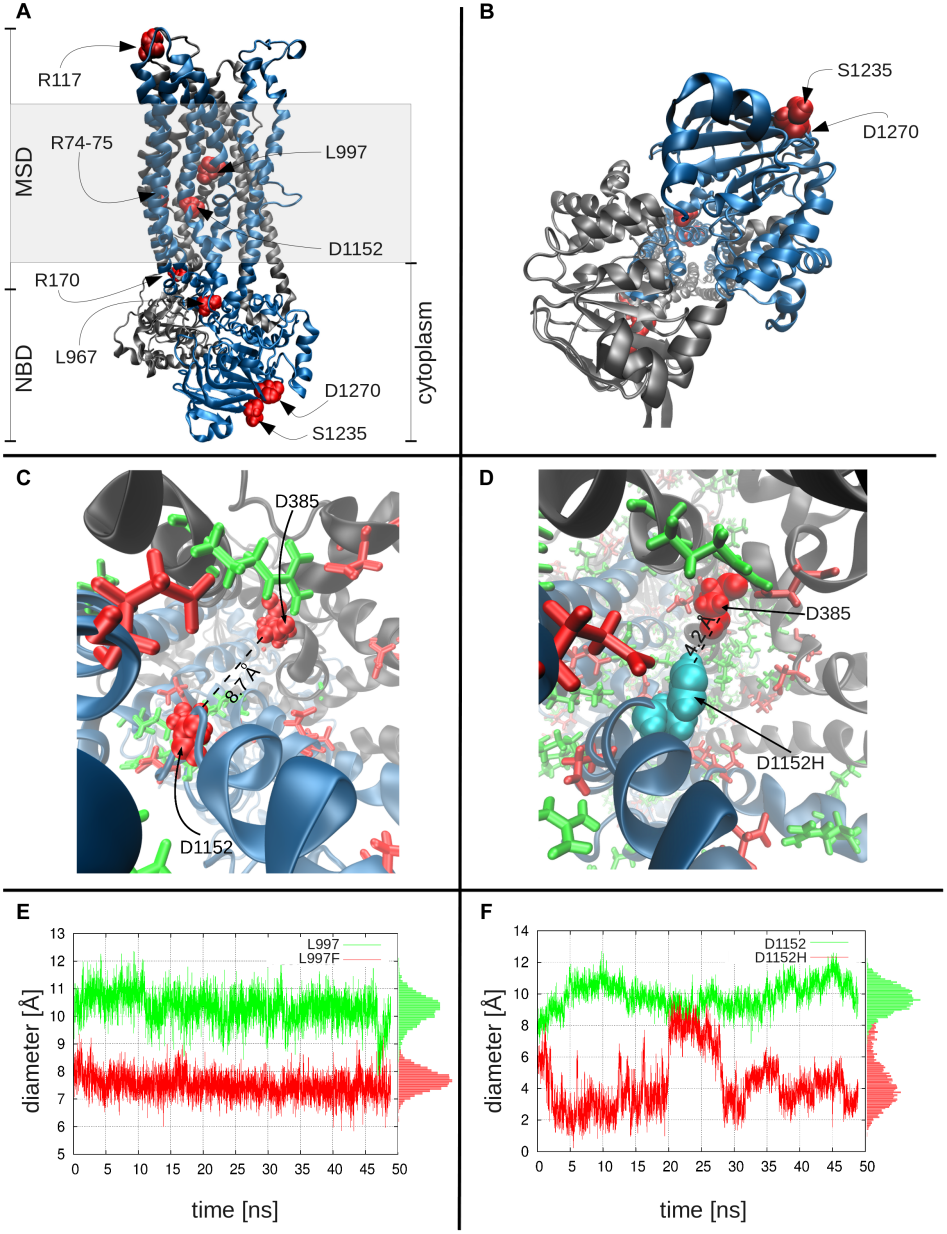
Molecular modeling and simulations of CFTR WT and variants. Panels **a** and **b** display the side and bottom views, respectively, of the WT CFTR dimer, where the two nucleotide-binding domains and the two membrane-spanning domains are labeled as NBD and MSD. The shaded region indicates the location of the lipid bilayer. **Color key**: *black*, subunit 1 of CFTR, with residues 1–859; *blue*, subunit 2, residues 860–1480; *red* CFTR variants studied. Panel **c** shows the charge distribution around D1152H: this negatively charged residue (*left*; shown in *red* space-filling representation) is surrounded by several positively charged residues (*green*), especially on its side of the cavity, creating an attractive force that keeps the residue from extending into the cavity. Also shown are other negatively charged residues (*red* stick or space-filling representation), including D385, diametrically opposite to D1152. Panel **d** shows the corresponding scene for the variant residue, D1152H (*cyan*), which can move toward the center of the cavity, thus leading to a constriction in the channel diameter. **Channel diameter at the location of variant residues:** Panel **e** shows the diameter of the channel at the location of L997, as a function of time, both for the WT (L997, *green* curve) and the variant (F997, *red* curve), based on closest interatomic distance between L997/F997 and D385. On panel **f**, the same information for the WT and variant D1152H is shown. In both plots, the pore diameter in the WT is larger than that stabilized in the mutants. The histograms of channel sizes are shown along the right ordinate.

The specific amino acid substitutions that interfere with WNK1/SPAK-activated transformation of CFTR to a more efficient bicarbonate-conducting channel are scattered throughout the linear DNA sequence, suggesting that three-dimensional structure and/or mechanisms of dynamic conformational changes linked to these amino acids are important risk for pancreatitis. We computationally modeled the molecular structure, and studied the dynamics, of wild type (WT) and mutated CFTR channels. Because the effective van der Waals radius of chloride (1.8 Å [34]) is smaller than that of bicarbonate (2.6 Å, see Methods), we tested whether amino acid substitutions that reduced the inner diameter of the CFTR channel could selectively impede bicarbonate conductance. A CFTR-WT model (**Figure 2a**) was constructed [35], [36] and used to locate and study *CFTR^BD^* functional variants (**Figure 1**). The model is based on the most recently resolved ABC transporter structure (from *Staphylococcus aureus* sav1866; see Materials and Methods). **Figure S3** **a** shows the superposition of our model on this crystal structure, which yields an RMSD of 1.6 Å. Panel **b** shows that residues lining the pore at the membrane-spanning domain (MSD), observed by the end of 50 ns simulations, agree in general with the CFTR model built by Norimatsu and collaborators [37], [38] which was also confirmed by cysteine scanning experiments [37], [39]. Likewise, the pore radius profile evaluated for our wild-type structural model (**Figure S3** **c** *solid curve*, with the gray band displaying the fluctuations observed in 50 ns simulations) is qualitatively consistent with that observed by Norimatsu and coworkers [37] for the MSD.

MD simulations comparing the channel diameters of the WT and mutants L997F and D1152H (**Figure 2c–f**) demonstrate that the channel diameter is observed to narrow down from an average value of 10.3 Å to 7.5 Å (standard deviation, σ = 0.5 Å) at the pore region, near the L997F amino acid substitution (**Figure 2e**), and from an average of 9.9 Å to 4.3 Å (σ = 1.1 Å) for the *CFTR^BD^* mutant D1152H (**Figure 2f**). Note that in contrast to the WT CFTR and L997F mutant where the structure maintains its stability, the D1152H mutation induces significant fluctuations in local conformation, which are reflected on the changes in the pore diameter at this location within the channel.

**Figure 3:**
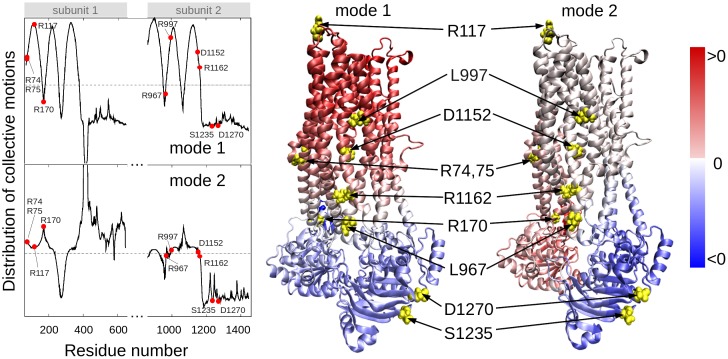
Location of selected variant sites with respect to the collective modes of the CFTR. The left panel displays the relative motions of individual residues along the collective modes 1 (*top*) and 2 (*bottom*) intrinsically accessible to the two transporter subunits (residues 71–645 and 846–1445, respectively). These modes divide the CFTR structure into two groups of residues (colored *red* and *blue* in the ribbon diagrams) subject to opposite-direction motions. The pale blue/pink indicate the central locations (or hinge regions) mediating the concerted anti-correlated movements of the two groups. L967, D1152 and R1162 participate in the hinge region that modulates the concerted anti-correlated (opening/closing) movements of the two membrane-spanning domains in mode 2. Residues indicated by filled points (*left* curves) or yellow spheres (*right* diagrams) are R74, R75, R117, R170, L967, L997, D1152, R1162, S1235 and D1270.

In order to determine residues that play a key role in the global dynamics of the CFTR, we performed an elastic network model (ENM) analysis. ENM analysis provides information on the mechanisms of collective movements intrinsically accessible to the structure, which usually enable structural changes relevant to function [40]. Application to CFTR highlighted the critical positioning of R74, R75, R170, L967, and R1162 at the hinge region that modulates the collective movements of the nucleotide-binding domains (NBDs) with respect to membrane-spanning domains (MSDs) (mode 1 in **Figure 3**). We also note that L967, L997, D1152, and R1162 act as anchors in collective mode 2. In this mode, the two NBDs are observed to move in opposite directions (see color-code diagram in **Figure 3**). The relative movements of the two NBDs, is known to control channel gating, hence the significance of this mode, or the alterations in mode 2 potentially caused by substitutions at the corresponding hinge site.

These two results suggest that substitutions of amino acids (or their side chains) at those particular regions could have an impact on the collective dynamics of CFTR, and interfere with concerted movements that would otherwise facilitate anion permeation. We noted that the mean-square fluctuations in our model are minimal at those particular residues (**Figure S4**), suggesting that mutations at those sites could not be accommodated without affecting the overall transporter structure and dynamics. Minimal mobility at those mutation sites originates from the contribution of global (most collective) modes. In contrast, the *CFTR^BD^* candidate variants D1270 and S1235 are in close proximity on the surface of the NBD2 (**Figure 3**), and had weaker functional effects than other *CFTR^BD^* variants (**Figure 1**).

### Association of CFTR^BD^ variants with sinus disorders and male infertility

To examine the potential clinical relevance of *CFTR^BD^* variants, we reviewed case report forms for additional CF phenotypic features of dysfunction in the sinorespiratory and male reproductive systems, which both use CFTR for bicarbonate secretion. Association with *CFTR^CF^* alleles was used to test for CFTR-mediated chloride secretion, *CFTR^BD^* to test for selective bicarbonate-mediated secretion and, because both *CFTR^BD^* and *CFTR^CF^* cause defective bicarbonate conductance, association with either *CFTR^BD^* or *CRTR^CF^* alleles, or recessive genotypes (*CFTR^BD^*/*CRTR^BD^* or *CFTR^CF^*/*CRTR^BD^*) to assess overall risk of altered bicarbonate secretion on organ dysfunction.

The sinuses may use CFTR bicarbonate secretion, in part, for mucus hydration [41]. Sinusitis is common, with a complex gene-environment-anatomic risk that includes anatomy, allergies and recurrent infections. Self-reported chronic sinusitis was more common in pancreatitis cases (n = 151; 15.9%) than in controls (n = 53; 10.2%, P = 0.002) (**Table 2**). We identified the R75Q, R117H, L967S, L997F, D1152H, and S1235R *CFTR^BD^* variants as well as *CFTR^CF^*-associated variants (e.g., F508del, G542X) in cases with rhinosinusitis. Sinusitis was reported in pancreatic cases who did not have any of the *CFTR* variants in our test panel (p = 0.021; OR 1.51; CI 1.05-2.18), but risk increased among carriers of *CFTR^BD^* (p = 0.001; OR 2.60, CI 1.43–4.60), *CFTR^CF^* (p = 0.01; OR 2.47; CI 1.18–4.91) or either *CFTR^BD^* or *CFTR^CF^* variant allele (p = 0.0001; OR 2.55; CI 1.55–4.15) (**Table 2**). Rhinosinusitis was not statistically associated with recessive genotypes, possibly due to the complex nature of chronic sinusitis or requirement for an unidentified epistatic risk factor.

CFTR bicarbonate secretion also plays a role in pH regulation in the male reproductive system[42]. Male infertility is uncommon and not dependent on recurrent infections. Self-reported male infertility over age 30 years was more common among cases (n = 17; 4.2%) than controls (n = 1; 0.4%, p = 0.03) (**Table 2**). We identified R75Q, R117H, and S1235R as well as the *CFTR^CF^* variants F508del, G542X and 2789+5G<A in male cases with infertility. There was no increased risk of male infertility in cases without *CFTR* variants (p = 0.28), but there was significant risk in cases with either *CFTR^BD^* or *CFTR^CF^* alleles (p = 0.023; OR 10.7; CI 1.03–536) or as a recessive genotype (p = 1.2×10^−7^; OR 303; CI 23–15783) (**Table 2**).

## Discussion

Our integrative approach revealed a new functional class of rare *CFTR* variants of clinical significance in pancreatic disease. Targeted genotyping of reported and plausible *CFTR* variants in our cohort identified candidate variants with a high pre-test probability of being diseases associated, and these were evaluated for specific functional studies in model cell types and focusing on a context-dependent signaling pathway. Although the *CFTR^BD^* variants were scattered throughout the genetic sequence, three-dimensional models of the protein provided insight into structural and dynamic mechanisms of dysfunction. Significant association between *CFTR^BD^* variants and symptoms of sinusitis and male infertility, but not overt lung disease as in CF, provided additional evidence of context-depended dysfunction in humans. We believe that this type of integrated approach will be important in understanding the genetic contribution to this and other complex disorders and informing the development of therapeutics that target the molecular etiology rather than the phenotype.

### Evidence that CFTR^BD^ variants are associated with pancreatitis

One of the challenges of genetic association studies is determining the effect of candidate genetic variants by statistical tests when the variant is rare or the mutation effect is uncertain. One approach is to increase study power by markedly increasing study subject numbers, but this approach is prohibitively expensive and not always feasible in rare diseases. Another approach is to evaluate the combination of statistical trends linked to studies of the functional effects of a variant in a biological system and a biologically plausible framework.

In the current study, 11 variants that were previously reported to be present in chronic pancreatitis but not CF causing [16], [17], [43]–[52] underwent functional testing. Only *CFTR* M470V and R1162 (not shown) did not meet criteria of altered bicarbonate permeability and/or conductance after WNK1 and SPAK activation (**Figure 1e–f**, discussed below). The remaining 9 *CFTR^BD^* variants were identified at least twice in pancreatitis association studies over the past decade.

Five variants (R74Q, R75Q, R170H, L967S, and R1162L) were located in the hinge region that modulates the collective movements of the NBDs with respect to the MSDs (**Figure 3**). R74Q was previously reported in a single chronic pancreatitis patient [53] but not in the CFTR2 database. *CFTR* R74Q was identified by us in two cases and no controls (p = ns) and in one case who was a *SPINK1* N34S carrier (p = 0.006). R75Q is considered to be a non-CF causing mutation according to the CFTR2 mutation database [54]. *CFTR* R75Q was identified in 61/906 cases and 75/1214 controls (6.3 vs. 6.2%, p = ns) but was also detected in nine *SPINK1* N34S/- mutation carriers (9/55, 16.4%), with strong combined effect (*SPINK1* OR 3.7, *SPINK1*+R75Q compound OR 12.2, p 0.002). Two of the nine trans-heterozygous cases had been previously reported[18]. R75Q was also identified in four cases with a concurrent severe CF-causing mutation and in no compound controls. R170H was first reported in two cases of congenital bilateral aplasia of vas deferens in England [53] but is not currently in the CFTR2 mutation database. *CFTR* R170H was identified in three cases and no controls (p = ns). L967S has been reported in a single case of azoospermia from the CF mutation database [53] but is not in the CFTR2 mutation database. L967S was identified in ten cases (one trans-heterozygote), two controls (OR 6.9 p = 0.004), and one N34S case carrier. R1162L is predicted to be a highly deleterious variant by SIFT and damaging by PolyPhen modeling [55] and is included in the CFTR2 mutation database and classified as a variant not causing CF. Although located in a critical portion of the CFTR molecule, the association and functional threshold for inclusion as a *CFTR^BD^* variant were not fully met.

Two variants (L997F and D1152H) appeared to reduce channel diameter. L997F is considered a mutation of varying clinical consequences for CF, with low rates of pancreatic insufficiency and retention of chloride conductance [54]. In this study L997F was identified both in the cases (0.7%) and controls (1.0%), additionally, L997F was identified in one N34S case carrier and three compound heterozygous mutation case carriers, but independent statistical association with pancreatitis was not demonstrated in this study. D1152H is a mutation of varying clinical consequence for CF and is associated with low rates of pancreatic insufficiency and retention of chloride conductance [53]. *CFTR* D1152H was identified in four cases and no controls (p = 0.014). Two of these cases were in compound heterozygosity with F508del.

Two variants (S1235 and D1270N) were on the surface of NBD2 (**Figure 2**). S1235 is a non-CF-causing mutation[54] and was identified in 2.4% of cases and 1.4% of control (p = ns), three compound heterozygous cases and one N34S case carrier. While this did not reach statistical significance in this cohort, multiple previous reports of CFTR S1235R in idiopathic pancreatitis patients[56], [57] and complex functional features [27] were noted. D1270N is of varying clinical consequences for CF, with low rates of pancreatic insufficiency and retention of chloride conductance [54]. D1270N was identified both in the cases (0.3%) and controls (0.2%). Although these variants have been identified in previous studies, the effects of these rare variants on altered bicarbonate permeability and conductance appear to be weak (**Figure 1** **e–f**) and the effect on the function of NBD2 (**Figures 2** **–** **3**) is unclear. However, they meet minimal criteria for the class on function grounds and contribute to the overall effect on disease risk.

The final variant (R117H) is located in an extracellular domain and has functional effects beyond the other *CFTR^BD^* variants. R117H is a complex variant that is associated with CF only when found in cis with a T5 tract in intron 8. The *CFTR* R117H variant was identified in 22 cases (2.3%) and 8 controls (0.7%) (p = 0.001), with only 3 cases and 1 control having the CF-associated R117H*T5 haplotype (p = ns), which links the *CFTR* variant R117H to pancreatitis regardless of the intron 8 T5 haplotype. R117H*T7/T9 was also identified in 9 of the 80 cases with a concurrent severe CF-causing mutation and in no CF carrier controls. The R117H variant was the only one with reduced chloride current density (**Figure 1b**). While the variant was associated with altered bicarbonate permeability and conductance, the mechanism is yet to be determined.

### Common CFTR variants previously associated with pancreatitis but not confirmed in the current study

The common polymorphisms M470V, T854T, and Q1463Q had no significant association with pancreatitis, either individually or combined in haplotypes, in contrast to a previous report [58]. Haplotypes were determined by counting homozygous carriers of each subset (M470V, T854T, P1290P, Q1463Q and M470V, IVS-T, IVS-TG) and applying Fisher's exact test. The IVS8 T/TG/M470V allele was evaluated in 784 NAPS subjects and controls, and no significant associations were found, in contrast to another report[59]. The possibility that a series of complex haplotypes affect CFTR expression or exon skipping was not excluded, but no evidence of direct association was seen in the current study or our previous pancreatitis GWAS [24].

Thirty-seven of the 81 *CFTR* variants tested were not identified in any cases among the NAPS2 cohort. The remaining variants were also not significantly overrepresented alone or with *SPINK1* or CF mutation carrier. I148T was seen in three cases and one control, so an effect could not be detected or excluded; the *in cis* deletion mutation 3199del6 was not detected in any I148T carriers. The IVS8T5 variant was identified in 9.9% of cases and 8.2% of controls, which is not individually significant. There were six N34S/T5 trans-heterozygote controls and no cases, but the combined odds ratio (OR 3.9) of the *SPINK1* N34S variant with IVS8T5 was not significantly higher than N34S alone. Four additional variants were identified in only one patient and no controls: CF mutations 2184delA, 3120+1G>A, R1162X and a mutation of varying clinical consequence, G1069R.

Taken together, these genotyping and functional studies provide strong rationale for inclusion of nine variants as *CFTR^BD^* class members. Although additional variants may be added to the *CFTR^BD^* class in the future, the current study did not have the very large patient size needed to provide adequate power to detect statistically significant changes in additional rare variants. In addition, other possible mechanisms of *CFTR* channel dysfunction linked to altered bicarbonate conductance are possible, such as mechanisms linked to *CFTR* R117H.

### Structural significance of CFTR^BD^ mutants

Structure-based simulations can provide insights into molecular driving forces and thereby into the mechanisms of channel dysfunction. To better understand the location and structural effects of the nine amino acid variants conferring risk of pancreatitis and causing dysfunction of the electrophysiological response to WNK1-SPAK activation, we developed structural models of CFTR and conducted dynamic simulations. Because no crystallographic structures for the entire human CFTR are currently available, we built a homology model based on the structure of a bacterial ABC transporter (Sav1866) from *Staphylococcus Aureus* [35]. Several computational studies have been carried out using models of CFTR and other ABC transporters that focus on the structure and/or gating cycle of the molecule and the effect of common mutations/deletions (e.g., F508del in CFTR) [4], [5], [35]–[37], [39], [60]–[64]. Our study is, to our knowledge, the first to investigate the multiscale dynamics of CFTR by examining both the global motions of the overall protein (with ENM) and the local effects of particular *CFTR^BD^* variants (with MD). The ENM analysis highlighted the critical positioning of R74, R75, R170, L967, and R1162 at the global hinge regions (those between the NBD and MSD of transporter in mode 1, and between the two NBDs in mode 2), as evidenced by the significant suppression of residue fluctuations in their close neighborhood. Mutations at those sites would thus be expected to interfere with the functional dynamics of the channel. Our all-atom MD study, on the other hand, showed that a substantial constriction could arise in channel diameter with substitutions at residues lining the wall of the channel. In particular, the L997F and D1152H mutants showed channel pore size reductions in their neighborhoods that would directly affect conductance properties.

### Defects in CFTR bicarbonate transport

The fact that all of the pancreatitis-associated variants identified by genetic screening in this study resulted in defective WNK1-SPAK-activted increase in bicarbonate secretion supports the argument that this mechanism is critical for bicarbonate-secreting cells that utilize CFTR as the primary anion channel. The importance of bicarbonate conductance across CFTR at the apical membrane is magnified if chloride, but not bicarbonate, conductance across the basolateral membrane is minimal, as predicted for the pancreatic duct cell [6], since the transcellular anion conductance is responsible for fluid secretion. Under basal conditions, CFTR-mediated bicarbonate permeability is only ∼20% of chloride, and the capacity for facilitating high bicarbonate flux for bicarbonate-secreting tissues is limited. Under conditions of low-intracellular chloride, the WNK1-SPAK pathway are activated, and this in turn transforms CFTR into a highly bicarbonate-permeable anion channel (**Figure 1**). The molecular mechanisms as to how WNK1-SPAK increases the CFTR bicarbonate permeability remain unclear. However, increasing evidence suggests that ion permeability of anion channels is not fixed and can be dynamically modulated by cellular signaling and other events [65].

The pore of anion channels is believed to have a large polarizable tunnel, where ion selectivity is basically determined by the hydration energy of ions and polarizability of the channel pore [65]. Therefore, in general, the CFTR ion channel is more permeable to large anions that are more readily dehydrated [66]. However, this cannot be applied to HCO_3_^−^. Although the size of HCO_3_^−^ (equivalent radius: 2.1 or 2.43 Å) is larger than Cl^−^ (1.81 Å), most anion channels, including CFTR, exhibit poor HCO_3_^−^ permeability because of the asymmetrical charge distribution of HCO_3_^−^ [67]. A decrease in the CFTR pore diameter, as shown in L997F, can affect the permeability of HCO_3_^−^ in many ways, such as by limiting the accessibility of large, asymmetrically charged HCO_3_^−^ to the channel pore. A second mechanism of reducing HCO_3_^−^ permeability and conductance is to inhibit the interaction between CFTR and WNK1/SPAK or to reduce the WNK1/SPAK-mediated conformational change of CFTR. The elucidation of precise molecular mechanisms of each mutation will provide insights into the understanding of HCO_3_^−^ conduction in CFTR and also in other anion channels.

### Conclusion

Taken together, these findings support a new class of CFTR functional variants with a specific defect in responding to WNK1-SPAK activation with increased bicarbonate permeability – conductance, dubbed *CFTR^BD^*. As a class, these nine variants are more common in pancreatitis cases than in controls and also had evidence of significant risk of pathology in other organs utilizing CFTR for bicarbonate secretion. New insight into multiple plausible mechanisms were gained by developing a structural model of the entire CFTR molecule and by analyzing the collective dynamics for wild type and disease-causing variants that result in altered channel function. Together, these findings provide new understanding of the complexity of pancreatic disease related to CFTR-associated duct dysfunction. Identification of members of this new class of *CFTR* variants on DNA sequencing of symptomatic patients in whom a bicarbonate channelopathy is suspected may provide insight into disease mechanisms and guidance for patient-specific clinical management decisions.

## Materials and Methods

### Study cohort

The NAPS2 cohort was ascertained, and data were collected as described previously [23]. All patients were prospectively enrolled using protocols approved by the appropriate IRBs. Physician-confirmed diagnosis of pancreatitis was required for enrollment as a case, while questions on CF, chronic sinusitis, and male infertility were included on a case report form administered by a clinical research coordinator. DNA and phenotypic data for patients with chronic and recurrent acute pancreatitis (n = 984) and healthy unrelated controls (n = 467 from the NAPS2 case-control study [23], [68] plus DNA from additional healthy controls from SomaLogic Inc. (Boulder, CO) (n = 377), the Inflammatory Bowel Disease Genetics Consortium (Dr Richard Duerr, University of Pittsburgh) (n = 338) and additional University of Pittsburgh studies of pancreatitis and pancreatic cancer (Drs David Whitcomb and Randall Brand, University of Pittsburgh) (n = 42) [24] were evaluated for a final study cohort of 984 cases and 1224 unrelated controls.

### Genotyping

*PRSS1* genotyping was done by DNA sequencing [69]. *SPINK1* genotyping was done by sequencing exons 2–3 and the flanking regions in a preliminary subset of 745 NAPS2 cases, with the entire cohort (cases and controls) genotyped for p.N34S, p.P55S and c.27delC using TaqMan assays. The *SPINK1* c.194+5G>A variant [70] was seen in one patient and one control; c.194+2T>C [71] was not identified in the initial sequencing and was not further genotyped.

CFTR variants for the screening panel were selected from a review of published papers and abstracts between 1998 and 2010 [16], [17], [43]–[52] and the open access CFTR mutation database based in the Hospital for Sick Children in Toronto (http://www.genet.sickkids.on.ca) and Johns Hopkins University (Http://CFTR2.org).

*CFTR* genotyping was done using a custom MassARRAY iPLEX Gold assay (Sequenom, Inc, San Diego, CA) or custom TaqMan Gene Expression Assays (Life Technologies Corporation, Carlsbad, CA) through the Genomic and Proteomic Core Laboratories at the University of Pittsburgh and verified by bidirectional DNA sequencing. All cases and controls were tested for each of the 81 selected *CFTR* variants (**Table S1**). Variants were selected in three stages: the most common CF-causing mutations in North America, variations that have been reported in pancreatitis literature at least once and a subset of variants that have been reported in CF patients but for which the biological and pathological relevance remains undetermined (Mutations of Undetermined Clinical Significance). 67 SNPs (125GtoC, 1716G>A, 1717-1G>A, 1898+1G>A, 2183AA>G, 2184delA, 2789+5G>A, 3120+1G>A, 3659delC, 3849+10kbC>T, 621+1G>T, 711+5G>A, A455E, D110H, D1152H, D1270N, D443Y, D579G, F1052V, F1074L, F508C, F508del, G1069R, G1244E, G1349D, G178R, G542X, G551D, G551S, I1131L/V, I148T, I336K/T, I507del, I807M, IVS8T5, K1180T, L1065P, L967S, L997F, M1V, M470V, M952I, M952T, N1303K, P67L, Q1463Q, R1070Q, R1162X, R117C, R117H, R170H, R258G, R297Q, R31C, R352Q, R553X, R668C, R74W, R75Q, S1235R, S1255P, S485R, S977F, T338I, T854T, V201M, W1282X) were multiplexed into 6 wells; 14 SNPs (S492F, S945L, R74Q, R560T, R1162L, G85E, I1027T, R334W, R347P, G576A, 711+1G>T, 1001+11C>T, P1290P, 3199del6) were ascertained separately via TaqMan Gene Expression Assays, with repeat confirmation of all positive results. 3199del6 was genotyped via TaqMan on all samples that tested positive for I148T. In addition, the intron 8 boundary was directly sequenced in 873 subjects to determine the significance of the IVS8 T/TG tract.

### Statistical analysis

Significant differences in carrier frequencies among cases and unrelated controls were determined by chi square analysis or Fisher's exact test, and two-tailed p-values are reported. The results of each set of experiments are presented as means ± SEM. Statistical analysis was performed using Student's *t-*tests or analysis of variance followed by Tukey's multiple comparison test as appropriate. *P*<0.05 was considered statistically significant.

### Cell culture and plasmids

HEK 293T cells were maintained in Dulbecco's modified Eagle's medium-HG (Invitrogen, Grand Island, NY) supplemented with 10% fetal bovine serum, 100 U/ml penicillin, and 0.1 mg/ml streptomycin. The mammalian expressible plasmids for hCFTR[72], Myc-rWNK1[21] and Flag-mSPAK [20] were described previously. Plasmids were transiently transfected into cells using Lipofectamine 2000 reagents (Invitrogen, Grand Island, NY). An average transfection rate over 90% was confirmed by transfection with a plasmid expressing green fluorescence protein (pEGFP-N1). Plasmids expressing variant hCFTRs were generated using a PCR-based site-directed mutagenesis kit (Stratagene, Santa Clara, CA).

### Immunoblotting

Immunoblotting was performed using conventional methods [20]. Briefly, cells were harvested with lysis buffer (20 mM HEPES pH 7.4, 150 mM NaCl, 5 mM EDTA, 1% Triton X-100, 1 mM NaVO4, and 1 mM β-glycerophosphate) containing a complete protease inhibitor mixture (Roche Applied Science, Mannheim, Germany). Protein samples were suspended in a sodium dodecyl sulfate buffer and separated by SDS-polyacrylamide gel electrophoresis. The separated proteins were transferred to a nitrocellulose membrane and blotted with appropriate primary and secondary antibodies, and protein bands were detected with enhanced chemiluminescence solutions. Antibodies against CFTR (M3A7, Millipore, Billerica, MA) and aldolase A (N-15, Santa Cruz Biotechnology, Inc., Dallas, TX) were obtained from commercial sources.

### Electrophysiology

Voltage and current clamp experiments were performed on HEK 293T cells transiently transfected with hCFTR as previously reported with slight modifications [20]. Briefly, cells were transferred into the bath mounted on a stage with an inverted microscope (IX-71, Olympus, Osaka, Japan). The pipettes were pulled by a Sutter P-57 puller and have free-tip resistances of about 2∼5 MΩ. These were connected to the head stage of a patch-clamp amplifier (Axopatch-700B, Molecular Devices, Sunnyvale, CA). Ag-AgCl reference electrodes were connected to the bath via a 1.5% agar bridge containing 3 M KCl. Liquid junction potentials were corrected for each experimental solution as described previously[20]. For the anion permeability test, individual data were corrected by measuring the offset potential shift induced by the replacement of anion solution after each experiment. The conventional whole-cell clamp was achieved by rupturing the patch membrane after forming a gigaseal. Voltage and current traces were stored and analyzed using Clampfit v.10.2 (Molecular Devices, Sunnyvale, CA). Currents were sampled at 5 kHz. All data were low-pass filtered at 1 kHz.

The high-chloride pipette solution contained (mM) N-methyl D-glucamine chloride (NMGD-Cl), 5 ethylene glycol tetraacetic acid, 1 MgCl_2_, 3 Mg-ATP and 10 4-(2-hydroxyethyl)-1-piperazineethanesulfonic acid (HEPES)(pH 7.2). The low-chloride pipette solution was prepared by replacing Cl^−^ with equimolar glutamate. The stand bath solution contained (mM) 146 NMDG-Cl, 1 CaCl_2_, 1 MgCl_2_, 10 glucose and 10 HEPES (pH 7.4). The high-bicarbonate-containing bath solution was made by replacing NMDG-Cl with equimolar choline-HCO_3_. The bicarbonate-containing solution was continuously gassed with 95% O_2_+5% CO_2_.

In all experiments, currents generated by CFTR were confirmed by the following three characteristics: 1) activation of current by the treatment with cAMP (5 µM forskolin and 100 µM 1-methyl-3-(2-methylpropyl)-7H-purine-2,6-dione (IBMX), 2) a linear I–V relationship and 3) inhibition of current by the treatment with the CFTR inhibitor CFTRinh-172.

The current reversal potential (E_rev_) was measured either in current clamp mode or in voltage clamp experiments. Resting membrane potential (RMP) was recorded in zero current clamp mode. To test the current-voltage relationship during zero-current clamp recording, clamp mode was shifted to the voltage clamp mode, and the I–V curve was achieved with ramp pulses from -100 to 100 mV (250 ms, holding potential; near the RMP). All currents were corrected for capacitative currents and the I–V relationship was plotted using the values of current density (pA/pF). The relative anion permeability was determined by the reversal potential shift (ΔE_rev_  =  E_rev_(X) – E_rev_(Cl)) induced by replacing extracellular Cl^−^ with X^−^ anion using the Goldman-Hodgkin-Katz equation as follows: P_X_/P_Cl_ =  exp(ΔE_rev_/(RT/zF) – ([Cl^−^]_o_/[Cl^−^]’_o_)) × ([Cl^−^]’_o_/[X^−^]_o_), where [Cl^−^]’_o_ is the bath concentration of Cl^−^; [Cl^−^]_o_ is the residual Cl^−^ in the substituted solution; [X^−^]_o_ is the concentration of substitute ion; and *R*, *T*, *z* and *F* have their conventional thermodynamic meanings. The anion outward chord conductance (G_X_: X is anion) between E_rev_ and E_rev_ +25 mV was achieved by linear plotting.

### Structural modeling

The homology model of human CFTR (UniProt accession code: P13569) was obtained using the Swiss-Model Workspace software [73]. The most recently resolved crystal structure of the *Staphylococcus Aureus* sav1866 ABC transporter, fitted to the human CFTR (PDB code: 4A82, 2.0 Å resolution) [36] was adopted as a template, and the structural model was completed using the X-ray crystallographic structure of the NMD2 region of human CFTR (PDB code: 3GD7, resolution 2.7 Å) (see **Figure S3a**). This model deviates from the template structure by 1.6 Å RMSD, and in our simulations the RMSD levels off at ∼3.5 Å. The MSD pore-lining residues and pore radius profile (**Figure S3b–c**) were consistent with those observed in a homology model constructed by Norimatsu and coworkers [37], [39], which was based on an earlier structure (PDB code: 2HYD, resolution 3 Å) [35]. Using this model for WT CFTR, we generated *in silico* models for the mutants L997F and D1152H.

Of note, the collective modes predicted by ANM are highly robust and they are not sensitive to small structural variations (like those due to a different model).

### Molecular dynamics simulations and elastic network model analysis

Molecular dynamics simulations were performed using the AMBER11[74] package (GPU version of the pmemd program), with the Amber99SB[75] force field and using the TIP3P water model. The protocol consisted of an initial minimization in vacuum, using 1,500 steepest descent and 1,500 conjugate gradient steps, to remove strong steric contacts, followed by another minimization of 5,000 steepest descent and 5,000 conjugate gradient steps, in explicit solvent, followed by a production run of 50 ns. The systems were kept at a temperature of 300 K, using Langevin dynamics with a collision frequency of 2 ps^−1^; the SHAKE algorithm was adopted to use a 2 fs time step. The stability of the system was assessed by verifying the convergence of the root mean square deviation (rmds) of its heavy atoms, after the first 5 ns of simulation.

As to the pore regions where we examined the local effects of substitutions, we allowed for the relaxation and optimization of interactions during the described protocol. The simulations, thus, gave rise to local rearrangements in the neighborhood of the mutation sites and permitted us to extract statistical data on the average pore diameter at the constriction zone and its fluctuations.

The elastic network model analysis of collective dynamics was performed using the approach reviewed earlier[40]. Collective modes of motions are evaluated by eigenvalue decomposition of the connectivity/Hessian matrix, using the Gaussian/Anisotropic network model. The shape of the mode permits us to identify regions subject to large fluctuations as well as domains undergoing anti-correlated movements (colored *blue* and *red* in the ribbon diagrams, **Figure 3**).

### Ionic diameter

The radii of the mono-atomic chloride ion was taken from Bondi [34]. The equilibrium geometry of bicarbonate ion was optimized using *ab initio* quantum mechanics at DFT level, with the B3LYP/6-311G** basis set, via the Gaussian 03 software. This resulted in a bicarbonate ion that could be fit in a minimum box of size 3.40 Å×4.86 Å×5.39 Å. This yields a van der Waals radius of 2.1 (or 2.43) Å for the bicarbonate ion, based on the two smaller dimensions (or the second largest dimension) that define the minimal cross-sectional area.

Unless specified otherwise, when we refer to the *diameter* of the pore, we mean the minimal diameter at the specific location of the mutation, as opposed to the distribution of diameters along the pore.

## Supporting Information

Figure S1 Measurement of P_HCO3_/P_Cl_ in cells without WNK1 and SPAK co-expression. Control experiments were performed. Whole-cell recordings were performed to measure CFTR bicarbonate permeability by replacing the bath solution with high HCO_3_^–^-containing (140 mM) solution. Cells were stimulated with cAMP (5 µM forskolin and 100 µM IBMX) after establishing whole-cell configuration. The current to voltage relationship (I/V curve) was obtained by depolarizing ramp pulses from −100 to +100 mV. The permeability ratio P_HCO3_/P_Cl_ was calculated according to the Goldman-Hodgkin-Katz equation. I–V relationships at the indicated points are presented in the right panel. The conductance ratio G_HCO3_/G_Cl_ was calculated by measuring each outward current (slope between E_rev_ and E_rev+25_ mV). Replacing the bath solution with a high-bicarbonate-containing solution induced a strong positive shift in E_rev_, indicating that bicarbonate permeability is much smaller than that of chloride. Summarized results of multiple experiments (n = 7) are presented in **Figure 1e and f**.
(PDF)

Figure S2 Representative current to voltage (I–V) plots of *CFTR* variants in whole-cell current measurements. Whole-cell recordings were performed to measure the CFTR HCO_3_^-^ permeability and conductance by replacing the bath solution with high HCO_3_^–^-containing (140 mM) solution. The pipette solution contained 10 mM Cl^-^. WNK1 and SPAK kinases were coexpressed with wild-type (WT) or variant CFTR. Cells were stimulated with cAMP (5 µM forskolin and 100 µM IBMX) after establishing whole-cell configuration. The I–V curve was obtained by depolarizing ramp pulses from −100 to +100 mV (250 ms), and all currents were corrected for capacitative currents. Treatment with the CFTR inhibitor CFTR_inh_-172 (20 µM) inhibited the HCO_3_^-^ currents by an average of 91.8±3.0% (WT-CFTR with WNK1 & SPAK coexpression, measured at +100 mV, n = 4) indicating that CFTR mediates most of the HCO_3_^-^ currents. The permeability ratio P_HCO3_/P_Cl_ was calculated according to the Goldman-Hodgkin-Katz equation. The conductance ratio G_HCO3_/G_Cl_ was calculated by measuring each outward current (slope between E_rev_ and E_rev+25_ mV).
(PDF)

Figure S3 Structural features of the present homology model and comparison with previous work. Panel **a** shows the superposition of the current homology model onto the X-ray structure of ABC transporter from *S. aureus*, which yields an RMSD of 1.6 Å. Panel **b** shows the position of some residues lining the pore at the MSD, located on two TM helices, 6 (*red*) and 12 (*yellow*). These residues were reported by Sansom and collaborators to be lining the MSD pore [61], based on both, a homology model and experimental cysteine scanning. Panel **c** shows the MSD pore radius profile—measured as the radius of the smallest circle that fits the cross sectional area at each elevation along the pore axis (perpendicular to the membrane plane), and its extension toward the cytoplasmic region. The MSD portion (indicated by the upper abscissa label) is comparable to that reported earlier by Norimatsu et al [37].
(TIFF)

Figure S4 Contribution of the slowest modes to the square displacements of residues in CFTR. Square displacements are calculated using the ENM representation of the two subunits of the structure (residues 71–645 and 846–1445, respectively). The right panel shows a color-coded ribbon diagram where regions subject to large fluctuations are colored pink, and those maximally constrained, blue. Note that R74, R75, R170, L967, D1152 and R1162 lie in the highly constrained region.
(TIF)

Table S1 81 CFTR variants genotyped in pancreatitis patients. The CFTR mutations investigated in this study are reported with legacy nomenclature and relative ranking among the American College of Medical Genetics most common classic cystic fibrosis-causing mutations found in North America (CF). Those found to be associated with cases in the current cohort include an X in the Panc Disease column. *IVS8 T5 and R117H are reported but CF disease causing only when *in cis* with each other or IVS8 T5 with IVS8 TG12or13. Intronic mutations are reported in standard nomenclature “####+/−##N>N” except IVS8-T5, (1210-12T[5]).
(DOCX)
