# Correction: Mechanisms of *CFTR* Functional Variants That Impair Regulated Bicarbonate Permeation and Increase Risk for Pancreatitis but Not for Cystic Fibrosis

**DOI:** 10.1371/journal.pgen.1004778

**Published:** 2014-10-06

**Authors:** The PLOS Genetics Staff

The alignment of the immunoblots in Figure 1A is incorrect. The authors have provided a corrected version here.

**Figure 1:**
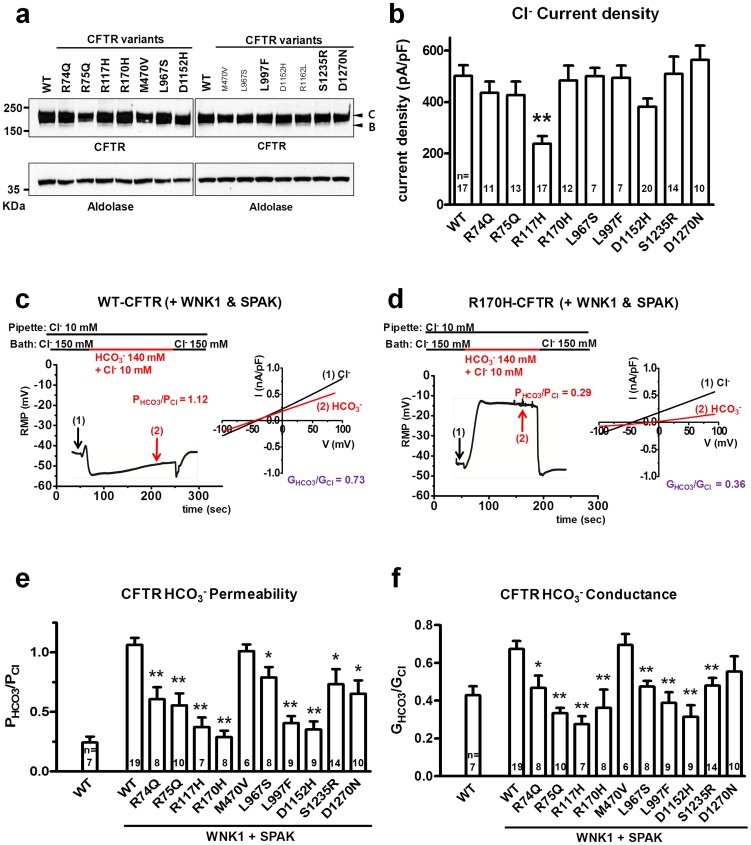
Functional characteristics of the nine *CFTR^BD^* variants. Panel **a**. Wild-type (WT) and variant CFTR proteins were expressed in HEK 293T cells and immunoblotted with anti-CFTR and anti-Aldolase antibodies. Replicate lanes are in small font. Band B, expected size of immature ER core-glycosylated CFTR; band C, mature complex-glycosylated CFTR. Panel **b**. Whole-cell Cl^−^ currents were measured in WT and variant CFTR-expressing HEK 293T cells, as described in Methods. Panel **c**. Whole-cell currents of WT-CFTR were measured in HEK 293T cells co-expressed with WNK1 and SPAK using patch pipette contained a low concentration of Cl^−^ (10 mM). A representative trace of reversal potential measurement is shown in the left panel. The permeability ratio P_HCO3_/P_Cl_ was calculated according to the Goldman-Hodgkin-Katz equation. I–V relationships at the indicated points are presented in the accompanying graph. The conductance ratio G_HCO3_/G_Cl_ was calculated by measuring each outward current (i.e., slope between E_rev_ and E_rev+25_ mV). RMP, resting membrane potential. Panel **d**. Whole-cell currents of R170H-CFTR were measured in HEK 293T cells using the same protocol shown in panel **c**. Panel **e**. A summary of the P_HCO3_/P_Cl_ values obtained from WT-CFTR in the standard state (left) compared to WT-CFTR and the nine *CFTR^BD^* variants with WNK1 + SPAK activation (right, underlined). Panel **f**. A summary of the G_HCO3_/G_Cl_ values in the standard state (left) with WNK1 + SPAK activation (right). Values throughout are means ± SEM. * p<0.05, **p<0.01: difference from WT in cells co-expressed with WNK1 and SPAK.
