# Supplementary figures and images for: Mechanisms of CFTR Functional Variants That Impair Regulated Bicarbonate Permeation and Increase Risk for Pancreatitis but Not for Cystic Fibrosis
Source: PLoS Genet. 2014 Jul 17;10(7):e1004376. doi: 10.1371/journal.pgen.1004376 (PMC4102440; doi:10.1371/journal.pgen.1004376)

# WT-CFTR (No WNK1 & SPAK)

Pipette:  $\text{Cl}^-$  10 mM

Bath:  $\text{Cl}^-$  150 mM  $\text{Cl}^-$  150 mM

$\text{HCO}_3^-$  140 mM  
+  $\text{Cl}^-$  10 mM

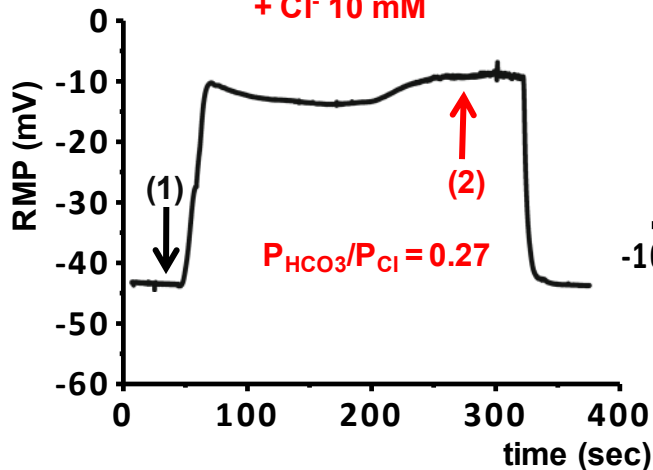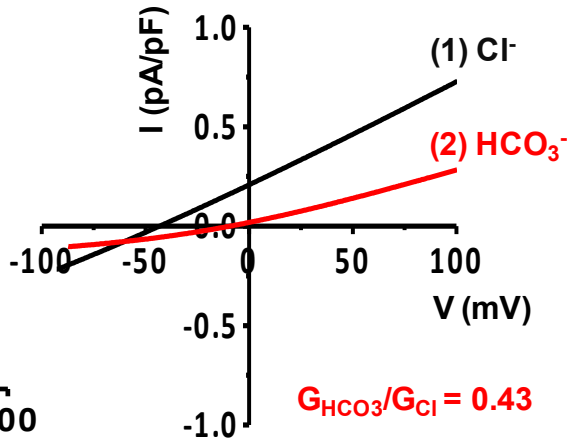

Supplement: Figure S1 — Measurement of PHCO3/PCl in cells without WNK1 and SPAK co-expression. Control experiments were performed. Whole-cell recordings were performed to measure CFTR bicarbonate permeability by replacing the bath solution with high HCO3 –-containing (140 mM) solution. Cells were stimulated with cAMP (5 µM forskolin and 100 µM IBMX) after establishing whole-cell configuration. The current to voltage relationship (I/V curve) was obtained by depolarizing ramp pulses from −100 to +100 mV. The permeability ratio PHCO3/PCl was calculated according to the Goldman-Hodgkin-Katz equation. I–V relationships at the indicated points are presented in the right panel. The conductance ratio GHCO3/GCl was calculated by measuring each outward current (slope between Erev and Erev+25 mV). Replacing the bath solution with a high-bicarbonate-containing solution induced a strong positive shift in Erev, indicating that bicarbonate permeability is much smaller than that of chloride. Summarized results of multiple experiments (n = 7) are presented in Figure 1e and f . (PDF) [file pgen.1004376.s001.pdf]

Figure S2

■  $\text{Cl}^-$   
■  $\text{HCO}_3^-$   
■  $\text{HCO}_3^- + \text{CFTR}_{\text{inh-172}} (20\mu\text{M})$

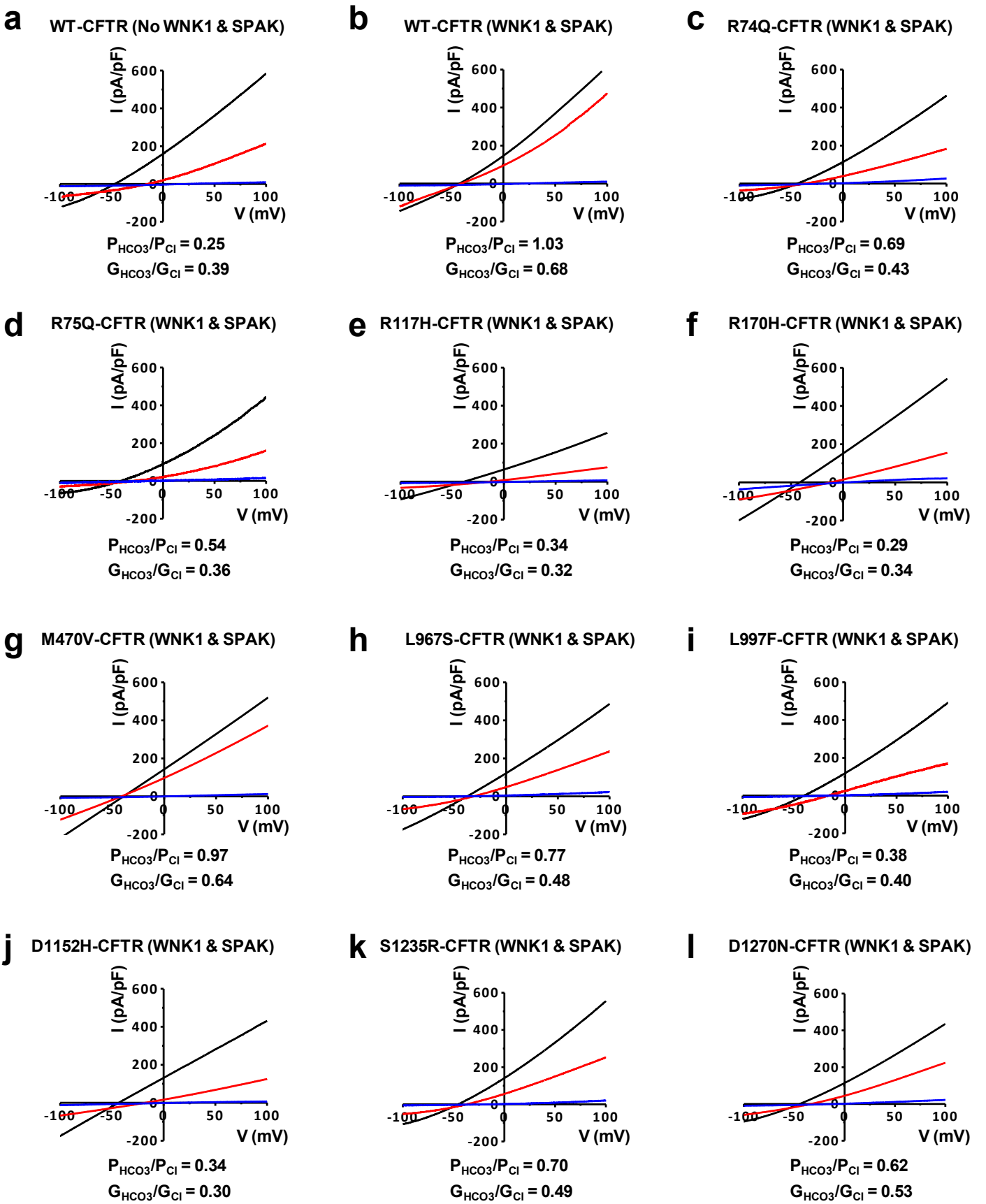

Supplement: Figure S2 — Representative current to voltage (I–V) plots of CFTR variants in whole-cell current measurements. Whole-cell recordings were performed to measure the CFTR HCO3 - permeability and conductance by replacing the bath solution with high HCO3 –-containing (140 mM) solution. The pipette solution contained 10 mM Cl-. WNK1 and SPAK kinases were coexpressed with wild-type (WT) or variant CFTR. Cells were stimulated with cAMP (5 µM forskolin and 100 µM IBMX) after establishing whole-cell configuration. The I–V curve was obtained by depolarizing ramp pulses from −100 to +100 mV (250 ms), and all currents were corrected for capacitative currents. Treatment with the CFTR inhibitor CFTRinh-172 (20 µM) inhibited the HCO3 - currents by an average of 91.8±3.0% (WT-CFTR with WNK1 & SPAK coexpression, measured at +100 mV, n = 4) indicating that CFTR mediates most of the HCO3 - currents. The permeability ratio PHCO3/PCl was calculated according to the Goldman-Hodgkin-Katz equation. The conductance ratio GHCO3/GCl was calculated by measuring each outward current (slope between Erev and Erev+25 mV). (PDF) [file pgen.1004376.s002.pdf]

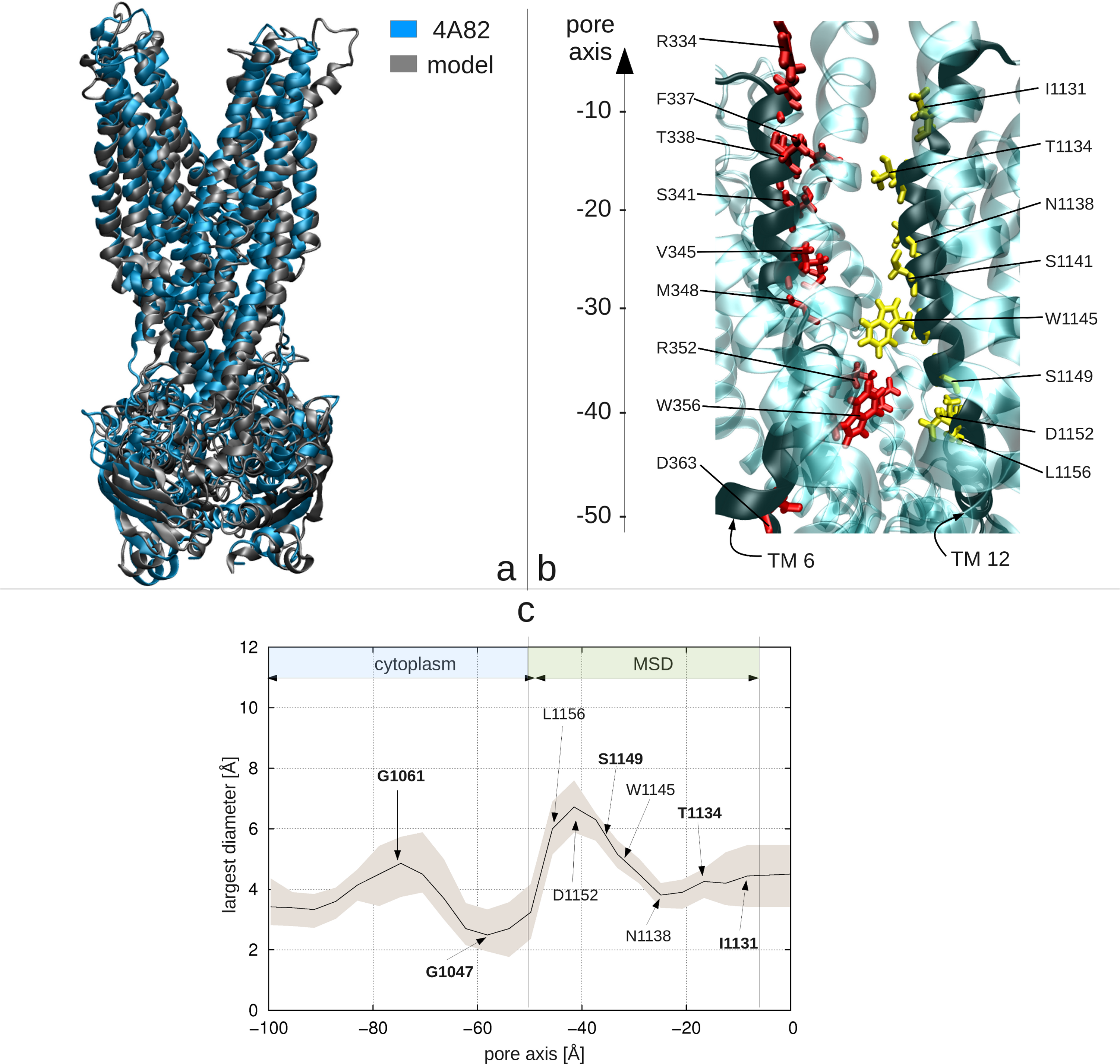

Supplement: Figure S3 — Structural features of the present homology model and comparison with previous work. Panel a shows the superposition of the current homology model onto the X-ray structure of ABC transporter from S. aureus, which yields an RMSD of 1.6 Å. Panel b shows the position of some residues lining the pore at the MSD, located on two TM helices, 6 (red) and 12 (yellow). These residues were reported by Sansom and collaborators to be lining the MSD pore [61], based on both, a homology model and experimental cysteine scanning. Panel c shows the MSD pore radius profile—measured as the radius of the smallest circle that fits the cross sectional area at each elevation along the pore axis (perpendicular to the membrane plane), and its extension toward the cytoplasmic region. The MSD portion (indicated by the upper abscissa label) is comparable to that reported earlier by Norimatsu et al [37]. (TIFF) [file pgen.1004376.s003.tiff]

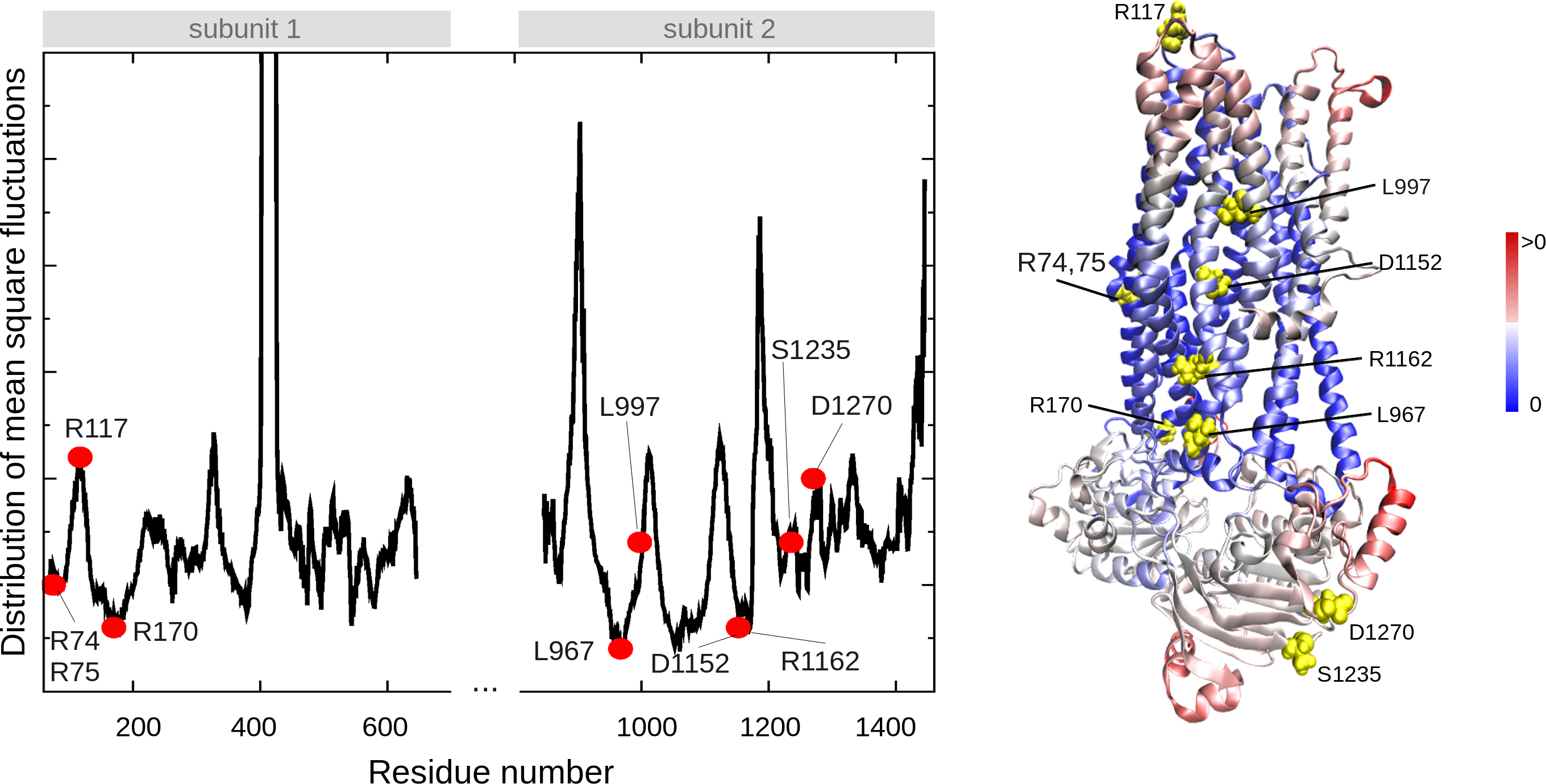

Supplement: Figure S4 — Contribution of the slowest modes to the square displacements of residues in CFTR. Square displacements are calculated using the ENM representation of the two subunits of the structure (residues 71–645 and 846–1445, respectively). The right panel shows a color-coded ribbon diagram where regions subject to large fluctuations are colored pink, and those maximally constrained, blue. Note that R74, R75, R170, L967, D1152 and R1162 lie in the highly constrained region. (TIF) [file pgen.1004376.s004.tif]
