# Supplementary material for: Mechanisms of CFTR Functional Variants That Impair Regulated Bicarbonate Permeation and Increase Risk for Pancreatitis but Not for Cystic Fibrosis
Source: PLoS Genet. 2014 Jul 17;10(7):e1004376. doi: 10.1371/journal.pgen.1004376 (PMC4102440; doi:10.1371/journal.pgen.1004376)
Supplement: Table S1 — 81 CFTR variants genotyped in pancreatitis patients. The CFTR mutations investigated in this study are reported with legacy nomenclature and relative ranking among the American College of Medical Genetics most common classic cystic fibrosis-causing mutations found in North America (CF). Those found to be associated with cases in the current cohort include an X in the Panc Disease column. *IVS8 T5 and R117H are reported but CF disease causing only when in cis with each other or IVS8 T5 with IVS8 TG12or13. Intronic mutations are reported in standard nomenclature “####+/−##N>N” except IVS8-T5, (1210-12T[5]). (DOCX) [file pgen.1004376.s005.docx]

**LaRusch et al.**

**Table S1**

| *CFTR mutation* | *CF*  *disease* | *Panc.*  *Disease* |
| --- | --- | --- |
| F508del | CF severe | X |
| G542X | CF severe | X |
| G551D | CF severe |  |
| N1303K | CF severe | X |
| W1282X | CF severe |  |
| R117H* | CF mild* | X |
| R553X | CF severe |  |
| 1717-1G>A | CF severe |  |
| 621+1G>T | CF severe | X |
| 2789+5G>A | CF mild |  |
| 3849+10kbC>T | CF mild |  |
| R1162X | CF severe |  |
| G85E | CF severe |  |
| 3120+1G>A | CF severe |  |
| I507del | CF severe |  |
| 1898+1G>A | CF severe |  |
| 3659delC | CF severe |  |
| R347P | CF mild |  |
| R560T | CF severe |  |
| R334W | CF mild |  |
| A455E | CF severe |  |
| 2184delA | CF severe |  |
| 711+1G>T | CF severe |  |
| IVS8 T5 | CF mild* |  |
| 125G>C |  |  |
| 1716G>A |  |  |
| 2183AA>G | CF |  |
| 711+5G>A | CF |  |
| D110H | CF |  |
| D1152H |  | X |
| D1270N |  | X |
| D443Y |  |  |
| D579G |  |  |
| F1052V |  |  |
| F1074L |  |  |
| F508C |  |  |
| G1069R |  |  |
| G1244E | CF |  |
| G1349D |  |  |
| G178R | CF |  |
| G551S |  |  |
| I1131L/V |  |  |
| I148T |  |  |
| I336K/T | CF |  |
| I807M |  |  |
| K1180T |  |  |
| L1065P | CF |  |
| L967S |  | X |
| L997F |  | X |
| M1V | CF |  |
| M470V |  |  |
| M952I |  |  |
| M952T |  |  |
| P67L | CF |  |
| Q1463Q |  |  |
| R1070Q |  |  |
| R117C | CF |  |
| R170H |  | X |
| R258G |  |  |
| R297Q |  |  |
| R31C |  |  |
| R352Q | CF |  |
| R668C |  |  |
| R74Q |  | X |
| R74W |  |  |
| R75Q |  | X |
| S1235R |  | X |
| S1255P |  |  |
| S485R |  |  |
| S977F |  |  |
| T338I | CF |  |
| T854T |  |  |
| V201M |  |  |
| 1001+11C>T |  |  |
| G576A |  |  |
| I1027T |  |  |
| P1290P |  |  |
| R1162L |  |  |
| R74Q |  |  |
| S492F | CF |  |
| S945L | CF |  |
